# Implementation, spread and impact of the Patient Oriented Discharge Summary (PODS) across Ontario hospitals: a mixed methods evaluation

**DOI:** 10.1186/s12913-021-06374-8

**Published:** 2021-04-17

**Authors:** Shoshana Hahn-Goldberg, Tai Huynh, Audrey Chaput, Murray Krahn, Valeria Rac, George Tomlinson, John Matelski, Howard Abrams, Chaim Bell, Craig Madho, Christine Ferguson, Ann Turcotte, Connie Free, Sheila Hogan, Bonnie Nicholas, Betty Oldershaw, Karen Okrainec

**Affiliations:** 1 OpenLab, University Health Network, Toronto, Canada; 2 Caregiver Advisor, University Health Network, Toronto, Canada; 3 Faculty of Medicine, University of Toronto, Toronto, Canada; 4 Toronto General Hospital Research Institute, University Health Network, Toronto, Canada; 5 Biostatistics Research Unit, University Health Network, Toronto, Canada; 6 Biostatistics Research Unity, University Health Network, Toronto, Canada; 7 Department of Medicine, Sinai Health System, Toronto, Canada; 8 Renfrew Victoria Hospital, Renfrew, Canada; 9 London Health Sciences Centre, London, Canada; 10 St. Josephs General Hospital Elliot Lake, Elliot Lake, Canada; 11 Markham Stouffville Hospital, Markham, Canada; 12 Thunder Bay Regional Health Sciences Centre, Thunder Bay, Canada; 13 Chatham Kent Health Alliance, Chatham, Canada

**Keywords:** Implementation, Discharge, Patient Centred, Transitions in care, Local adaptability, Triangulation, Patient experience, Quality improvement, Hospital

## Abstract

**Background:**

Traditional discharge processes lack a patient-centred focus. This project studied the implementation and effectiveness of an individualized discharge tool across Ontario hospitals. The Patient Oriented Discharge Summary (PODS) is an individualized discharge tool with guidelines that was co-designed with patients and families to enable a patient-centred process.

**Methods:**

Twenty one acute-care and rehabilitation hospitals in Ontario, Canada engaged in a community of practice and worked over a period of 18 months to implement PODS. An effectiveness-implementation hybrid design using a triangulation approach was used with hospital-collected data, patient and provider surveys, and interviews of project teams. Key outcomes included: penetration and fidelity of the intervention, change in patient-centred processes, patient and provider satisfaction and experience, and healthcare utilization. Statistical methods included linear mixed effects models and generalized estimating equations.

**Results:**

Of 65,221 discharges across hospitals, 41,884 patients (64%) received a PODS. There was variation in reach and implementation pattern between sites, though none of the between site covariates was significantly associated with implementation success. Both high participation in the community of practice and high fidelity were associated with higher penetration. PODS improved family involvement during discharge teaching (7% increase, *p* = 0.026), use of teach-back (11% increase, *p* < 0.001) and discussion of help needed (6% increase, *p* = 0.041). Although unscheduled healthcare utilization decreased with PODS implementation, it was not statistically significant.

**Conclusions:**

This project highlighted the system-wide adaptability and ease of implementing PODS across multiple patient groups and hospital settings. PODS demonstrated an improvement in patient-centred discharge processes linked to quality standards and health outcomes. A community of practice and high quality content may be needed for successful implementation.

**Supplementary Information:**

The online version contains supplementary material available at 10.1186/s12913-021-06374-8.

## Background

Traditional discharge summaries are laden with clinical information meant for the primary care provider. Instructions for patients are often not patient-centered or not included [1]. Moreover, while approximately 80–90% of hospitals in Ontario have a discharge planning process, previous reports suggest it is not standardized throughout the province, where involvement of the patient in this process and education provided is not consistent [2]. Patient-centered discharge tools, unlike traditional discharge summaries, engage the patient and family during the hospital admission with an individualized discharge plan and are associated with improved patient outcomes [3–6]. While improving care transitions is a focus of health systems globally, no Canadian tools exist to our knowledge. Very few tools from other countries have been co-designed with patients and families to address barriers to understanding and use of their discharge instructions [7, 8].

**Fig. 1:**
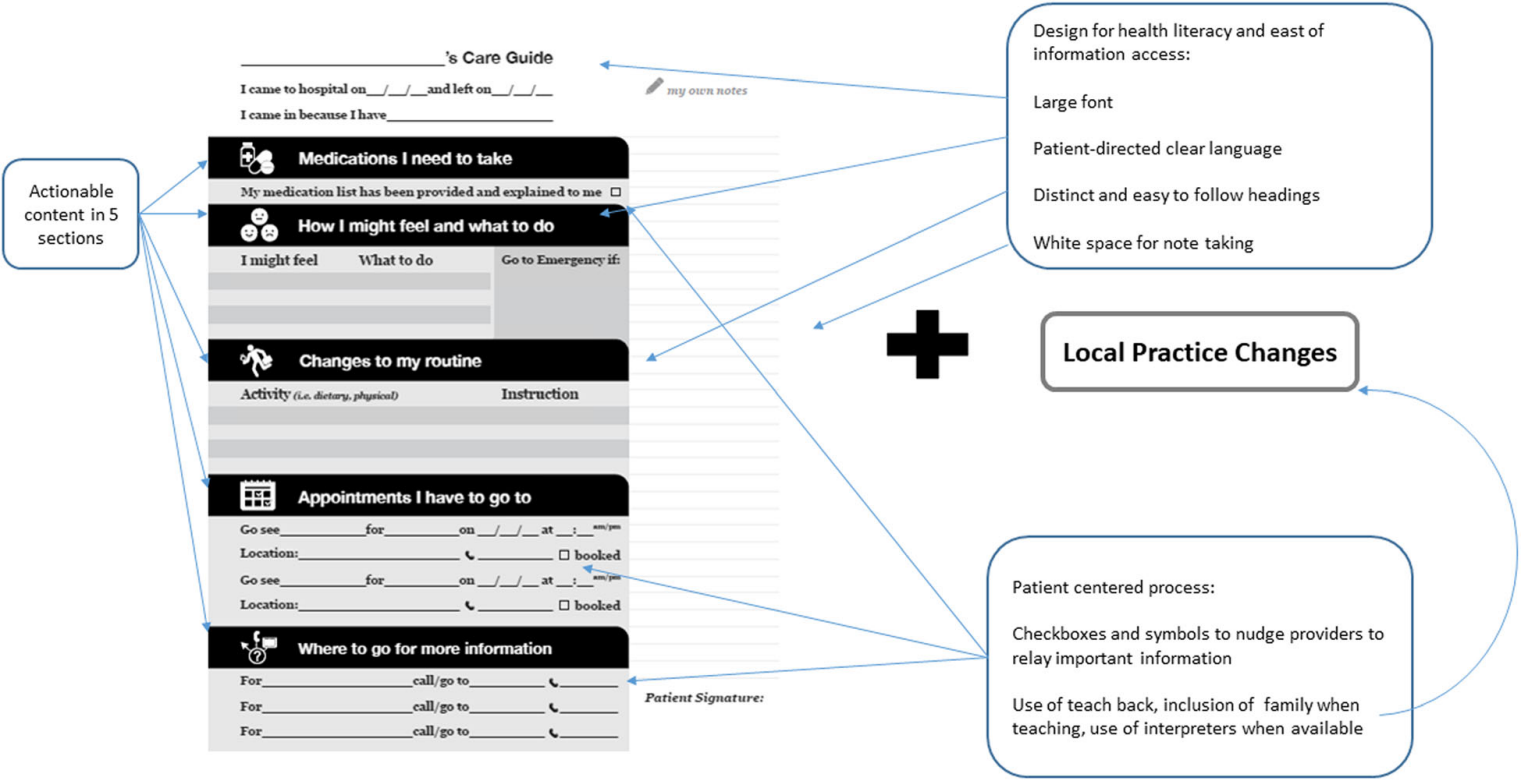
Image of the basic PODS template. Content and design features are identified and associated local practice change guidelines are listed

The Patient Oriented Discharge Summary (PODS) is an individualized discharge tool designed with patient and family engagement that contains five sections of information that are actionable and useful for patients and families; 1) medications, 2) changes to daily activities and diet, 3) follow-up appointments, 4) resources for patients and families, and 5) expected and worrisome symptoms to watch out for after leaving hospital (Fig. 1) [1]. PODS utilises design features such as plain language, large fonts, pictograms, and white space for patients to take notes that enhance retention and understanding of information [9]. PODS includes accompanying process guidelines which highlight key patient-centered processes proven to increase adherence to instructions [10, 11]. PODS is designed to be modular. Every organization that implements it is tasked with adapting the tool and guidelines to meet the needs of their patients and internal processes for discharge using a quality improvement process.

Other complex discharge interventions with some overlapping components to those in the PODS such as Project RED in the United States [12, 13] and the TRANSITION tool in Australia [14] have been successfully implemented and show promising improvements in patient experience, patient understanding, and even hospitalizations. However, studies evaluating the system-wide implementation of these complex interventions are lacking. This is especially true of evaluations of these interventions at larger scales inclusive of multiple diverse organizations, making it difficult to assess whether they are adaptable and transferable to other settings, described as phase IV by Campbell et al. [15] in their framework for evaluation of complex interventions in healthcare. While a pilot study in Toronto by our group demonstrated that PODS was both usable and feasible and suggested improvements in patient understanding of discharge instructions and patient experience, implementation and evaluation were limited to short-term pilot analysis of patient understanding and satisfaction [16]. The present study, of a large provincial implementation enables the study of a combination of different measures at the system level.

### Aims

In the present work, we aimed to understand 1) implementation of PODS in different settings and factors influencing increased penetration and 2) the effectiveness of PODS on patient-centred discharge processes, patient and provider experience, and 30-day emergency department (ED) visits and readmissions.

## Methods

### Setting and context

Ontario is Canada’s largest province with a population of more than 14 million people and 123 acute care and 58 rehabilitation hospitals, spread across 14 Local Health Integration Networks (LHINs), designated geographical areas. The hospitals are commonly grouped as being rehabilitation or acute care and as being academic, community, or small hospitals [17, 18]. This project was the result of an Accelerated Research to Improve Care (ARTIC) grant, government funding for province-wide quality improvement (QI) implementation of evidence based initiatives [19]. Following the consolidated framework for implementation research (CFIR), [20] factors known to facilitate successful implementation were built into both the processes of identifying participating hospitals and supporting the implementation. As part of the project application process, hospitals were selected based on the presence of an executive sponsor and a clear rationale for implementing PODS. Hospitals were chosen from all 14 LHINs and from within all hospital categories to be representative of the province as a whole.

Of 42 hospitals that applied, 21 were invited to participate in a community of practice (COP) and received a stipend to support their implementation and data collection. The hospitals involved all had a high level of organizational commitment and readiness as defined by the identification of an executive sponsor, clear rationale for implementing PODS in line with strategic goals, previous quality improvement experience, and sufficient resource capacity for project management. However, the hospitals ranged widely in size, geographic area, target patient population, discharge process (i.e. what members of the healthcare team were involved in providing patient education at discharge), whether PODS was implemented in isolation or as part of broader discharge process improvements, and whether the process was supported through the electronic medical record (EMR). There were eighteen acute care and three rehabilitation hospitals, eight which were considered academic hospitals, five large community hospitals and eight small community hospitals with under 100 beds (Table 1).

**Table 1:** Description of hospital and implementation characteristics of hospitals implementing PODS (*n* = 21 hospitals)

ID	Hospital characteristics		Implementation characteristics		
	Type	Size	Target population	Main Responsible Provider	When is it done
**1**	A	A	Mental health inpatients	Social work or nurse	Week of discharge
**2**	A	A	all inpatients	Physician	week of discharge
**3**	A	A	all medicine acute and sub-acute	Physician	week of discharge
**4**	A	A	all inpatients	Multidisciplinary	week of discharge
**5**	A	A	medicine, chronic disease, oncology, surgery	Nurse	day before discharge
**6**	A	A	Medicine - focus on elderly	Team	day of discharge
**7**	A	C	all surgery	Nurse	over whole stay
**8**	A	C	mental health ED and inpatient followed by rehab	nurse	week of discharge
**9**	A	C	all inpatients and ED (QBPs at first)	nurse	day before discharge
**10**	A	C	Surgery	nurse	day before discharge
**11**	A	S	medicine, surgery, and rehab	nurse	day before discharge
**12**	A	S	medicine, surgery, and ED	nurse	week of discharge
**13**	A	S	all inpatient (target CHF, COPD, and Stroke at first)	nurse	week of discharge
**14**	A	S	all inpatients and ED	nurse	day before discharge
**15**	A	S	all inpatients	nurse	week of discharge
**16**	A	S	all geriatric inpatients	nurse	week of discharge
**17**	A	S	medicine, surgery, and obstetrics	nurse	day before discharge
**18**	A	S	medicine, surgery, obstetrics	nurse	week of discharge
**19**	R	A	all inpatients (rehab includes stroke)	social work or nurse	over whole stay
**20**	R	A	inpatient Stroke	nurse	over whole stay
**21**	R	C	Rehab including stroke	nurse	Week of discharge

*Type: A* acute, *R* rehab, *Size: A* academic, *C* community, *S* small

Hospitals are categorized by type (acute or rehabilitation) and size (small with under 100 beds, community with over 100 beds yet no embedded research, or academic with over 100 beds and embedded research). Implementation characteristics describe the target population for the intervention, who delivers the intervention and when the intervention is delivered

Our team supported the implementation of PODS at the 21 hospitals in three stages: (1) Start Up: hospitals were guided through the process of adapting the PODS tool and process together with stakeholders; (2) Plan Do Study Act (PDSA): hospitals “went live” with either a pilot group or their full target group and then iteratively tested, refined, and evaluated the tool and process while implementing; and (3) Scale Up: hospitals spread the use of the tool and mentored others who were interested. Using a community of practice model, [21] where all the project teams came together for collective learning and sharing while implementing PODS, our team hosted regular meetings for education, shared knowledge and mentorship. A website was developed to house central resources and collective knowledge [22]. Project teams were encouraged to engage with patients and families throughout the project. A project advisory group was formed to guide the project with representation from the hospital, community, and patients including authors (SHG, TH, AC, HA, CB, KO). The group provided advice on project implementation, evaluation, and interpretation of results.

The project started in April 2017, with hospitals beginning implementation of PODS between April 2017 and March 2018. Majority of hospitals “went live” in October and November of 2017. Evaluation data was collected between April 2017 and December 2018 and spanned all three stages of start up, when hospitals had yet to discharge any patients with the PODS, and PDSA and scale up, when hospitals expanded the number of patients receiving the intervention. Implementation generally took between 3 and 6 months and required the support of a project lead with some amount of dedicated time. Once the PODS is implemented, although quality improvement techniques are recommended to refine and evaluate the intervention, no additional resources were required.

### Study design

Due to variation in adaptation and implementation across organizations, we chose to use an effectiveness-implementation hybrid design that uses both quantitative and qualitative methods for evaluating complex interventions [23]. Specifically, we employed mixed methods to achieve our first aim of understanding implementation while also addressing our second aim, of assessing the effectiveness of PODS. According to the categories of outcomes outlined by Proctor et al. [24], implementation was measured using implementation outcomes of acceptability, penetration, and fidelity and effectiveness was measured by assessing whether there were improvements in both service (process) and client (patient, family, and provider) outcomes (see Table 2). Service outcomes included measures of efficiency and patient centeredness and client outcomes included measures of satisfaction, function, and symptomatology.

**Table 2:** Characteristics of outcome measures categorized within the framework presented by Proctor et al. [23] of implementation, service, and client outcomes. Information includes description of measures and datasets and variables used for each category

Category	Measures	Dataset	Variables
**Implementation Outcomes**			
Acceptability	Experience of implementation as part of the community of practice	End of project interviews (qualitative)	Description of experience
Penetration	Proportion of eligible target patients who received the intervention over time	Quarterly data submissions from hospitals End of project interviews (qualitative)	% of target discharges who received a PODS Description of penetration patterns
Fidelity	Consistency – how often the intervention is used with eligible patients Completeness – when used, is the full intervention used with all its components Content Quality – is the content of the form patient centred and using plain language Process Quality – is the caregiver involved and is teach back used while delivering the intervention	End of project interviews (quantitative and qualitative)	Mean project lead ratings out of 10 on consistency, completeness, content quality, and process quality Qualitative explanations provided during interviews
**Service Outcomes (Process Outcomes)**			
Efficiency	Workload of providers delivering the intervention	Pre-post surveys of providers (Question asked in post survey only)	Y or N response to the question of whether the intervention adds to the workload Open comments related to the Y or N response provided via the survey comment field
Patient-Centeredness	Use of teach back while delivering the intervention Involvement of caregivers while delivering the intervention Discussions with patients about whether they had the help they needed at home	Pre-post surveys with providers Pre-post surveys with patients and families	Rating out of 10 of how often teach back is used Rating out of 10 of how often caregivers are included Y or N to whether there was a discussion about help needed at home before discharge
**Client Outcomes (Patient, Family, and Provider Outcomes)**			
Satisfaction	Perceived value of the intervention for providers Perceived value of the intervention for patients and families	Pre-post surveys with providers (Question asked in post only) Pre-post surveys with patients and families (Question asked in post only)	On both surveys: Y or N to whether the intervention added value. Qualitative explanations provided in the comment field
Symptomatology	Patient understanding of their medications and of what to do if concerned after leaving hospital	Pre-post surveys with patients and families	Likert scale rating of understanding of medications and of understanding of what to do if concerned after leaving the hospital
Function	Return ED visits and readmissions within 30 days	Quarterly data submissions from hospitals	All cause 30 day ED visits within the target group All cause 30 day readmissions within the target group

### Data collection

Data was collected via four data sets: (1) Hospital submissions of quarterly data describing their target population and intervention implementation; (2) pre-post surveys (Additional File 1) with providers involved in the discharge process; (3) pre-post surveys (Additional File 1) with a random sample of patients and families at each hospital (a different sample was collected after discharge for pre- and post-implementation of the PODS tool); and (4) end-of project interviews with project leads from each hospital.

The surveys were created by the project team with specific questions related to the intervention and outcomes of interest. Whenever possible, questions were taken from the Canadian Institute for Health Information Patient Experience Survey for Inpatient Care, a validated survey used after discharge at many Canadian hospitals [25–27]. In total, three questions were taken from that survey, one of which is also questions used in the US HCAHPS survey by the Centers for Medicare and Medicaid Services and the Agency for Healthcare Research and Quality, which are validated and used extensively in the United States. The questions used have a Cronbach alpha above the cut-off of 0.65 (unpublished data, Canadian Institute for health Information and L Russell, 2018).

Qualitative data was collected throughout the project through multiple sources which included: surveys, presentations at community of practice meetings and open-ended responses in both provider and patient and family surveys. The themes that emerged through the data were used to create a semi-structured interview guide developed specifically for this project (see Additional File 1) that was then used to conduct end-of-project interviews over the phone with project teams from each of the 21 hospitals. Interviews were conducted by authors SHG (PhD) and CM, who were coordinating the CoP and are trained in qualitative research methods. The purpose of collecting the qualitative data was known to the project teams and was collected to inform the implementation outcomes related to each hospital’s implementation process and barriers and facilitators impacting the implementation guided by the CFIR framework [20]. Interviews were between 30 and 60 min. They were recorded and transcribed for analysis.

#### Implementation outcomes

Implementation data was collected through quarterly submissions of penetration within the target population. Context and explanations of the experience of implementation through the community of practice model was collected during end-of-project interviews. Additionally, during the interviews, project teams were asked to rate the quality of their implementation using measures of consistency, completeness, quality of the content of their PODS, and quality of their process of providing PODS to patients in order to measure fidelity.

Factors impacting implementation were collected through demographic data on the target populations reported quarterly by hospitals and through information about the organization and their discharge process collected during end-of-project interviews with each project team (see Additional File 1 for the interview guide). Factors were chosen based on whether they were thought of as important by the project advisory team, whether they were present in the literature on implementation success, or whether they were found as a theme that emerged through qualitative data collected throughout the project.

#### Service (process) outcomes

Provider survey measures included the use of teach-back methods and involvement of family during discharge education, in order to see if using PODS effected important and evidence-based patient-centred processes. Additionally, providers reported whether the intervention added to workload as a measure of efficiency. Patient and family surveys included a question on whether patients had a discussion before discharge about the help they would need at home, another measure of patient-centeredness. Several questions within the surveys had open comment fields so that participants could add context and explanations to their responses.

#### Client (patient, family, and provider level) outcomes

Providers and patients were both surveyed on whether the intervention added value to the discharge experience. Patient and family survey measures also included a rating of understanding of medications, and a rating of understanding of what to do if they are worried about their condition, t. These specific questions, along with the service question about the discussion about help needed at home were chosen because they are relevant questions related to transitions in care selected by the Canadian Institute for Health Information Patient Experience Survey for Inpatient Care, and endorsed by provincial, Canadian and international quality agencies, based on their use in the Hospital Consumer Assessment of Healthcare Providers and Systems (HCAHPS) survey to measure quality of care transitions. Quarterly submissions from hospitals included data on all-cause 30-day return ED visits and readmissions for the target population.

### Analysis

Penetration was evaluated by measuring the proportion of patients in the target population who received a PODS in each quarter relative to implementation. To this end, a generalized linear mixed effects model was used, with fixed effects for quarter relative to implementation and between site covariates, and random slopes and intercepts for each site to model within site temporal trends. The mean age, percent male, and percent of the population with language barriers were included in the model as fixed effects.

Exploratory analysis using a non-parametric Wilcox Rank Sum test was conducted to examine any association between patient, hospital, implementation process, fidelity, and provider-reported outcome factors aggregated to the hospital level with whether a hospital had high penetration within the target population (defined as 75% or more of target patients receiving a PODS by Q4 after implementation). Factors were chosen as supported by literature and the qualitative findings.

Numerical summaries of provider, patient, and family reported data on patient-centred processes, patient understanding, and discharge experience were assessed by comparing means before and after implementation using generalized estimating equations (GEE) to account for within site clustering of responses [28, 29]. Change in healthcare utilization was analysed by plotting change over time. Linear mixed effects regression was used to assess percentage of 30-day return ED visits and readmissions, with percent PODS, within site, as the predictor of interest.

The analyses and data visualizations were performed using R version 3.6.2 by John Matelski and supervised by George Tomlinson (Biostatistics Research Unit, UHN).

Qualitative data was analysed using an iterative constant comparative process involving descriptive and interpretive analyses, open-coding, and identifying themes in the data. Study leads and members of the research team (SHG, TH, CM), read transcripts, then met to discuss initial codes and develop a preliminary coding framework. Interpretation of themes was discussed among team members to achieve consensus. Theoretical saturation, constant comparative analysis, trustworthiness, and validity checks provided assurance of data quality and rigor.

Using a triangulation approach, qualitative and quantitative results were interpreted together, towards the understanding of factors that may influence successful implementation and to draw inferences from the data.

This study was approved by the University Health Network Research Ethics Board.

## Results

### Implementation outcomes

The mean age of the target population across all hospitals was 66.4 ± 13.5 years, 48.7% ± 7.8% were male, and 7.8% ± 8.8% had a language barrier. Rehabilitation hospitals had the lowest (average of 80) target population discharges per quarter and academic hospitals had the largest number of discharges with an average of 813 per quarter.

All hospitals felt successful in their implementation. The most common barriers to implementation cited were competing priorities within the hospital, limited resources, and changes in project staff. Hospitals felt supported in working to overcome these barriers through the community of practice and by working together with their executive sponsor.

Between April 2017 and December 2018, there were 65,221 discharges within the target populations and 41,884 patients received a PODS (64.2%). Penetration as a percentage of target discharges increased over time, reaching 78% of the target population by the end of the study. The odds of penetration in each quarter relative to implementation also increased according to the generalized linear mixed effects model, from 2.90 in the second quarter to 24.29 in the fifth quarter. In addition, 12 sites had unplanned spread with a total of 25,190 patients outside the target groups receiving a PODS.

**Fig. 2:**
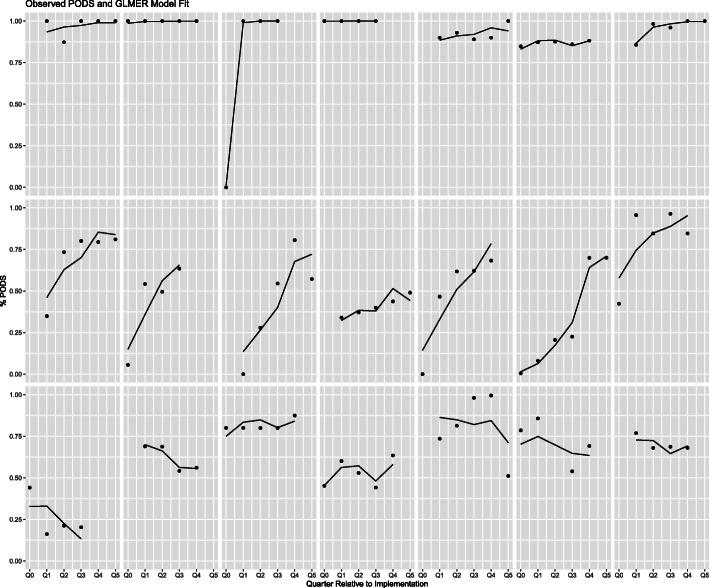
Individual hospital penetration via a generalized linear mixed effects model presented as percent of target by quarter after implementation (i.e. Q1 is the first quarter after implementation). Q0 refers to hospitals who began implementation in the middle of a quarter (i.e. a portion of the quarter includes data from before implementation began)

Penetration patterns varied widely from site to site (Fig. 2) with three common patterns: (1) Seven hospitals reached nearly 100% of the target population by the second quarter after implementation with majority reaching high penetration almost immediately following implementation, (2) seven hospitals slowly increased coverage within the target population, and (3) seven hospitals had a dip in implementation as their original target group was increased to a larger group.

Hospitals varied in their implementation patterns for several reasons. Findings showed that hospitals identified that 100% reach early was either due to size, as it was easier for smaller hospitals to implement a change in practice across all patient groups simultaneously, or due to implementation within an EMR, as some EMRs required mandatory PODS completion, enforcing a completed PODS for every patient discharged. A majority of hospitals that had a slower increase or dip in their implementation pattern identified initially utilizing PDSA cycles with small groups then slowly expanding to their full target group. Many hospitals identified trialing PODS with small groups at first facilitated implementation because it increased staff engagement and integration of PODS into the frontline process.

Measurement of fidelity was high across institutions. Average quality scores ranged from 8.1 for both acute and community hospital categories to 9.3 for small hospitals (Table 3).

**Table 3:** Fidelity as measured by ratings out of 10 provided during end-of-project interviews by project leads from each hospital

Measure	All Hospitals	Acute	Rehab	Academic	Community	Small
**Consistency**	8.6	8.7	8.3	8.6	8.3	10.0
**Completeness**	8.0	7.6	8.6	8.1	7.9	8.6
**Content Quality**	8.5	8.2	8.8	8.8	8.3	10.0
**Process Quality**	8.0	7.9	8.0	8.1	7.9	8.7
**Average**	8.3	8.1	8.4	8.4	8.1	9.3

Consistency in how often PODS was used, whether use was complete by using all elements of the intervention, the quality of the content in terms of patient centredness and use of plain language, and the quality of the process in terms of use of teach back and involvement of caregivers during delivery of the intervention

All hospitals identified a need for continual work to improve implementation quality. Even for hospitals that implemented with nearly 100% of their target population early on, they continued to work on improving quality, as can be seen from the following quote:

*“We have to watch and train on using it properly and the use of plain language and ongoing PDSA is needed. We do audits and are providing feedback to the providers via practice hints. This is one of the ways that we augment the PDSA cycle.”* (Project lead)

Very few patient, hospital, implementation process, fidelity or provider-reported factors showed association with a high level of penetration as defined as penetration within the target group of 75% or more by the fourth quarter after implementation (Table 4). Variables were all aggregated to the hospital level to assess association with hospital penetration. Trends showed association between higher penetration with higher ratings of quality (fidelity) and high participation in the community of practice.

**Table 4:** Results of exploratory analysis using a non-parametric Wilcox Rank Sum test to uncover group differences/associations based on levels of penetration of the intervention (high penetration is defined as 75% or more by Q4 after implementation)

**Variable**	**High Median**	**Low Median**	***p*-value**
**Continuous Variables**			
**Patient demographics**			
Age of the target population (mean)	63.5	68.2	0.915
Percentage of the target population that is male	45.0	50.0	0.166
Percentage of the target population with a non-English preferred language.	6.5	4.0	1.000
**Fidelity ratings (high is a score of 7 or higher out of 10 and low is under 7)**			
Project lead rating of consistency	9.2	8.5	0.146
Project lead rating of completeness	8.2	7.5	0.297
Project lead rating of content quality	10.0	8.0	0.018
Project lead rating of process quality	8.0	8.0	0.248
**Service and client outcomes reported by providers on post-implementation surveys**			
Mean workload (% Yes)	0.7	0.8	0.832
Mean value (% Yes)	0.9	0.9	0.304
**Categorical Variables**			
**Variable**	**High Percent**	**Low percent**	***p-value***
**Hospital categorization**			
Hospital Category			1.000
Academic	41.7	33.3	
Community	25.0	22.2	
Small	33.3	44.4	
Hospital Type			0.229
Acute	75.0	66.7	
Rehabilitation	0.0	33.3	
**Implementation process characteristics**			
Community of Practice participation			0.080
high (attendance at over 75% of meetings)	75.0	66.7	
medium (attendance between 25 and 75%)	25.0	0.0	
low (attendance at under 25%)	0.0	33.3	
Electronic Medical Record integration			1.000
Yes	41.7	44.4	
No	58.3	55.6	
Implementation as part of broader QI			1.000
Yes	58.3	55.6	
No	41.7	44.4	
Multidisciplinary team involved in delivery			0.331
Yes	83.3	55.6	
No	16.7	44.4	

Variables are all aggregated to the hospital-level and include patient demographics, hospital categorization, differentiating characteristics of each hospital’s implementation process, fidelity ratings provided by projject leads during end-of-project interviews, and service and client outcomes reported by providers through post-implementation surveys

Hospitals reported many factors that facilitated implementation. In general, these fell into three categories: (1) the intervention, (2) the implementation process, and (3) the organization.

Factors about the intervention that facilitated implementation were adaptability of the tool to local context of various patient groups and unique processes within the same hospital, co-design of PODS included patient and family input, and unplanned and natural spread. Local adaptability was described as being particularly important, as is highlighted in the following quote:

*“We adapted because our patients have a high amount of dementia. We wrote it in a way that a caregiver or loved one could read it. That way it’s not given directly to the patient and the caregiver is looped into the discussion.”(Project lead)*

Factors about the process of implementation that facilitated implementation were strong engagement with multi-disciplinary stakeholders, integration with frontline processes via a thorough current state assessment including removing alternative workflows and building the tool into the EMR if applicable, and participation in the community of practice, as is highlighted in the following quote:

“*We benefited greatly from having a platform for collaboration and learning from other sites during the implementation phase which was hosted by the lead project team. Being part of the group allowed us to secure strong leadership support and dedicate resources to patient and family engagement.”(Project lead)*

Organizational-level factors that facilitated implementation were having a commitment to the implementation, smaller size hospitals, and integrating implementation into other QI initiatives. Smaller hospitals identified that it was easier to coordinate the project and engage and educate staff. Senior management in smaller hospitals, who wear “*multiple hats*”, found it easier to be more involved. Larger hospitals found it helpful to create small pilot projects and then expand to other departments. Integrating PODS into other QI initiatives was identified as both a facilitator and a consequence of implementation, as described in the following quote:

*“PODS was part of a bigger discharge push. We leveraged our Nursing Workflow Optimization project (an informatics project) because PODS rollout and nursing workflow are well aligned. We also developed several new inputs that helped with implementing PODS [...] All of these new inputs were piggybacked off of PODS [...] Instead of being a product of optimization PODS helped drive optimization.”(Project lead)*

### Service and client outcomes

Hospitals surveyed 627 providers and 324 patients/families pre-implementation. 348 providers and 173 patient/families completed post-implementation surveys. A majority (64.1%) of providers felt that PODS added to their workload, however 86.2% found that it added value to the experience of providing discharge instructions. These measures of efficiency and satisfaction were not correlated with high penetration (Table 4). 98% of patients and families reported that PODS added value to their discharge experience.

Many patients and families added explanations and comments within their surveys on the usefulness of PODS, noting that they referred to the form at home and found having all the information in one place valuable. Some patients felt that it was not necessary for them, but that it would be especially useful if “*you don’t have a lot of healthcare experience*.” Those with previous experience in the same hospital commented on the improvement from previous forms. Several patients said it would have been more useful if provided earlier during their stay.

Many providers added comments on the usefulness of PODS for patients and families. Many also identified that it was only useful when the whole care team collaborated on completing the document and several providers commented that it enhanced collaboration.

**Fig. 3:**
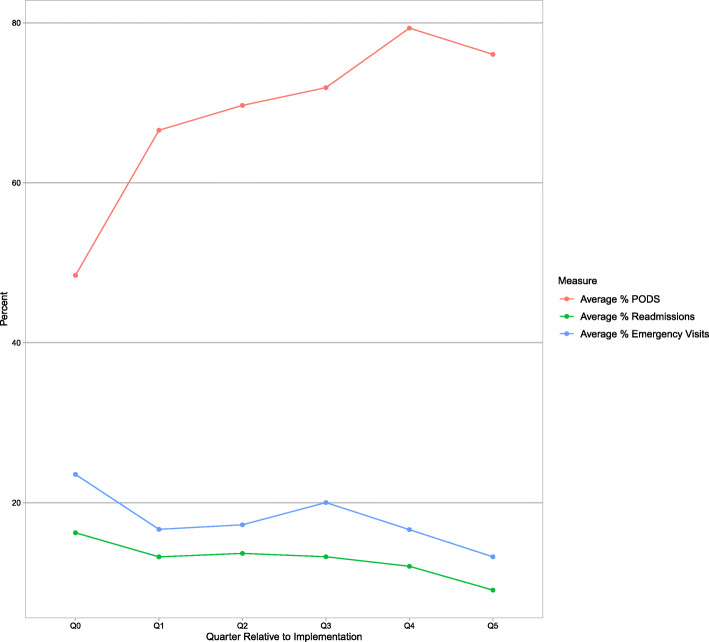
Quarterly all-cause 30-day readmissions and ED visits within the target groups plotted together with PODS penetration

Caregiver presence, use of teach-back and discussion about help needed at home improved with PODS implementation (Table 5). Over time, as sites increased penetration and fidelity, readmissions and ED visits decreased (Fig. 3). However, there was no significant change in readmissions or ED visits as a function of penetration (Table 5).

**Table 5:** Service and client outcome results. Efficiency and satisfaction outcomes are presented as the overall mean. Patient centeredness and symptomology outcomes are calculated using GEEs. Function outcomes are modeled using linear mixed effects regression

Variable	Pre/Post	Mean	***P*** value
**Service Outcomes (Process Outcomes)**			
**Efficiency**			
**Provider-reported addition to workload (percentage yes)**	Pre	–	–
	Post	0.64	
**Patient Centredness**			
**Provider-reported caregiver presence (scale out of 10)**	Pre	6.30	0.026
	Post	7.00	
**Provider-reported use of Teach Back (scale out of 10)**	Pre	5.40	< 0.001
	Post	6.50	
**Patient-reported discussion about Help needed at home**^a^ **(percentage yes)**	Pre	0.86	0.041
	Post	0.92	
**Client Outcomes (Patient, Family, and Provider Outcomes)**			
**Satisfaction**			
**Provider-reported value added (percentage yes)**	Pre	–	–
	Post	0.86	
**Patient and family reported value added (percentage yes)**	Pre	–	–
	Post	0.98	
**Symptomatology**			
**Patient-reported understanding about medications**^a^ **(scale out of 3)**	Pre	2.51	0.26
	Post	2.56	
**Patient-reported understanding of what to do if worried**^a^ **(scale out of 3)**	Pre	2.42	0.654
	Post	2.49	
**Function**			
	**% (95% CI)**		
**Change in return all-cause 30-day ED visits for a 10% increase in % of target population receiving a PODS**	−0.068 (−0.546–0.410)		0.776
**Change in return all-cause 30-day ED visits for a 10% increase in % of target population receiving a PODS**	−0.372 (−0.233–0.977)		0.224

^a^questions 19, 37, and 38 of the CIHI CPES-IC survey [24]

*GEE* generalized estimating equations

Within their added comments, many providers specifically noted the value of the teach-back process. The majority of providers who identified increased workload associated with PODS also noted qualitatively that the increase was small but worthwhile given the value added, as supported by the following quote:

*“It increases workload a little, but it is worthwhile because it helps me to feel more confident that the patient know what to do and expect when they get home.” (Nurse)*

## Discussion

Over the course of the project, many diverse Ontario hospitals succeeded in implementing the PODS intervention with variation in penetration and implementation pattern between sites. High participation in the community of practice and high fidelity ratings were associated with higher penetration. PODS met a need to improve standardization and quality of patient-centred discharge processes, which was supported by a growing patient-centred focus across hospitals and policy circles [8].

### Implementation outcomes

As expected, consistent with the CFIR framework, [20] having organizational commitment and a structured community of practice with a process of iteration and stakeholder involvement, were important to increased penetration. This was also reflected through qualitative reports identifying how the community of practice and executive sponsor helped in overcoming common barriers. To this end, our team, together with the hospitals involved in this project, developed an online community of practice open to anyone interested in implementing PODS. Future hospitals interested in implementing PODS should ensure executive commitment and consider joining the online community of practice or developing their own communities with other hospitals or within their hospital.

Local adaptation of the PODS tool was found to be a facilitator in this project and is known to play an important role in the diffusion of innovations [30, 31]. Likewise, local adaptation is considered the most important intervention characteristic within the CFIR [20]. The majority of individualized discharge tools are not developed through a process of co-design with patients and families [7]. The co-design process used to develop PODS was an important facilitator to implementation because organizations valued the source of the intervention and felt confident in the strength and quality of the tool. This also occurs when hospitals feel confident in the quality of their PODS, consistent with the association between high fidelity and penetration. These factors are intervention characteristics identified in the CFIR [20].

Small hospital size was an identified facilitator. This is inconsistent with implementation frameworks, [20, 32] which find larger organizations more likely to be successful predominantly due to stability of the organization and having sufficient resources to run the project. Interestingly, the CFIR notes several features of an organization’s internal setting as important for implementation success that are consistent with the smaller organizations in our cohort, namely having intertwined networks and good communication. Additionally, all hospitals, regardless of size, had to ensure sufficient resources for implementation before joining the project. Although our data did not show association between hospital size and penetration, smaller hospitals had higher fidelity highlighting the importance of combining penetration and fidelity when evaluating implementation success.

There were many factors studied that did not have any association with penetration including patient age, patient sex, whether the patient had a language barrier, whether the hospital was acute or rehabilitation, and whether the providers found that it added to their workload. These were factors that we would have thought might make the PODS more difficult to implement, however seem to be offset by the value added by the tool and by adapting the tool to make it part of the frontline process, meeting the needs of patients, families, and healthcare providers.

### Service and client outcomes

The PODS intervention was found to be effective in improving patient-centred processes, specifically involving the family, using teach-back, and having conversations about help needed at home. These findings are especially important in light of a recent Ontario-wide study, [33] which found that family involvement in discharge planning and knowing who to call with questions about your condition, both key features of PODS, were two of the top five priorities identified to help improve discharge experience. The remaining top responses in the study focused on lack of available homecare supports. Although PODS does not address homecare capacity, it facilitates a discussion of the help available or needed by patients at home prior to discharge.

As a balancing measure and in line with evidence, [34] we know that provider workload and perceived value are important measures for adoptability of QI initiatives such as PODS. We found that providers indicated that PODS did increase their workload, yet added value to the discharge experience and neither factor on its own was correlated with increased penetration. This was confirmed with qualitative data, which showed that the added value was enough to offset the minimal addition to workload. This also fits with the CFIR, [20] which identifies the perceived advantage to implementing a tool as a factor supporting implementation.

Unscheduled healthcare utilization decreased over the course of the project, however no connection was found between the percentage of the target population receiving a PODS and healthcare utilization. The lack of connection can be partially explained by the earlier identified need to look at both penetration and fidelity to assess success. Over time, with both implementation reach and improved quality due to improving PODS quality and other QI initiatives, there was a decrease in resource utilization. Similarly, a review of interventions using the ideal transitions in care framework, [35] found that although the majority of interventions designed to improve transitions do not result in reduced utilization, having multiple interventions concurrently addressing different elements of transitions results in the best chances of reducing readmissions.

### Strengths and limitations

This project has strengths as a large-scale implementation was evaluated that included many diverse hospitals representative of the Ontario-wide landscape. Large scale quality improvement implementations are rarely described in the literature [36]. By studying the implementation of PODS, we are able to understand how the community of practice was helpful and why organizations implemented the intervention in different ways. It provided insight into how future quality improvement communities of practice might be structured to support the needs of all participants. It also provided insight into interpretation of effectiveness results.

There were limitations with how hospital data on resource utilization was reported. Data was collected on whole target populations and not collected specific to patients who received PODS. Logically, this data still provides a useful estimate of resource utilization because the percentage of the target population who received a PODS is known and consistently increased over time. For survey data collected from providers, patients, and families, there was clustering because of variation in the number of responses each hospital was able to obtain and clustering in the actual site level means of the response variables. This is a limitation in terms of predicting effectiveness for particular sites, yet is a strength in that the estimated effects pool over a diversity of sites. For our analysis, we used this strength and aggregated the data and did not use it to infer organization-specific conclusions. Additionally, there was approximately 5% missing data in survey responses. At this level, we would expect to find significant effects using the GEE methods used. The addition of qualitative methods to triangulate and analyze results was also used as a method of minimizing these limitations. Lastly, this project was not designed to study causal inferences and designs with individual patient or cluster randomization are needed to study a causal link between PODS and health outcomes including adherence to discharge advice, healthcare utilization, and whether there are specific patient groups that are more likely to benefit.

## Conclusions

This project confirmed the adaptability and ease of implementation of the PODS tool and highlighted the usefulness of implementing PODS to improve patient-centred discharge processes, a need and growing focus among hospitals internationally. In particular, we found that quality of PODS content and participation in a community of practice were factors that should be considered by hospitals implementing PODS and similar interventions in the future. Future research in the field of implementation of healthcare QI should consider studying hospital size, which was found to be a facilitator to implementation that differs from general implementation frameworks. Our findings can help inform policy related to hospital transitions as well as, more generally, future system-wide collaborative implementation projects centered on patient-centered tools where different implementation patterns and practices are prevalent.

## Supplementary Information

**Additional file 1.** Interview Guides and Surveys Developed for this Project. The file includes one interview guide and two sets of survey questions with descriptions of response options and the sources of the survey questions. The interview guide was developed and used to interview project leads at the end of the project. The first survey, Patient/Caregiver Survey Question Information, was provided to patients and caregivers at two points in time, pre- and post-intervention. The second survey, Provider Survey Information, was provided to healthcare providers at two points in time, pre- and post-intervention. These materials were used to collect some of the qualitative and quantitative data presented in the manuscript.

## Data Availability

The datasets used during the current study are available from the corresponding author on reasonable request.

## Abbreviations

PODS: Patient Oriented Discharge Summary
ED: Emergency Department
ARTIC: Accelerating Research to Improve Care
QI: Quality Improvement
CFIR: Consolidated Framework for Implementation Research
EMR: Electronic Medical Record
COP: Community of Practice
PDSA: Plan Do Study Act
GEE: Generalized Estimating Equations
UHN: University Health Network
