# Supplementary material for: Implementation, spread and impact of the Patient Oriented Discharge Summary (PODS) across Ontario hospitals: a mixed methods evaluation
Source: BMC Health Serv Res. 2021 Apr 17;21:361. doi: 10.1186/s12913-021-06374-8 (PMC8052788; doi:10.1186/s12913-021-06374-8)
Supplement: Supplementary file 1 — Additional file 1. Interview Guides and Surveys Developed for this Project. The file includes one interview guide and two sets of survey questions with descriptions of response options and the sources of the survey questions. The interview guide was developed and used to interview project leads at the end of the project. The first survey, Patient/Caregiver Survey Question Information, was provided to patients and caregivers at two points in time, pre- and post-intervention. The second survey, Provider Survey Information, was provided to healthcare providers at two points in time, pre- and post-intervention. These materials were used to collect some of the qualitative and quantitative data presented in the manuscript. [file 12913_2021_6374_MOESM1_ESM.docx]

**Additional File 1: Interview Guides and Surveys Developed for this Project**

**Semi-structured interview guide for project teams:**

Estimated Time: 30-60 min

Thank you for making time to talk to us today. Myself and my colleague will be conducting the interview today. This close out interview is going to help us learn in more detail about your experience implementing PODS. In particular, we will be asking you questions related to themes that have come up over the course of our community of practice as important towards implementing PODS and it’s sustainability in the long term.

- Think back to why you chose to do PODS in the first place. Now that PODS is implemented, has it met those needs?
- Did anything about your hospital effect implementing (help/hurt) PODS?
- Were you successful in implementing in your target groups?
- Have you integrated with or leveraged other ongoing initiatives in or outside of your organization?
- Tell us about the key stakeholders that have given support to your project and the impact or importance that has had on the success or sustainability.
- Are you using a paper or electronic version of PODS? What are the advantages and disadvantages of using the one that you’re using?
- Can you tell us about how PODS is integrated into your discharge processes?
- If you had to give your organization a rating between 1 and 10, where 10 is the best on the following:
  - Consistency in using PODS
  - Completeness of the PODS being used
  - Quality of the content in the PODS
  - Quality of the process used
- Are there specific patient groups or areas within your hospital that needed special consideration when implementing?

**Patient/Caregiver Survey Question Information**

| **Question** | **Response Options** | **Source** |
| --- | --- | --- |
| Are you a patient or a caregiver? (A caregiver could be a family member, a friend, or a paid helper) | Patient  Caregiver | Project team |
| How long has it been since your most recent hospital discharge? | Within a week  More than a week but less than a month  More than a month but less than 3 months  More than 3 months | Project team |
| Did anyone at the hospital talk to you about what you would need to take care of yourself at home? | Yes  No | CPES-IC and  HCAHPS |
| Before you left the hospital, did you have a clear understanding about all of your prescribed medications, including those you were taking before your hospital stay? | Not at all  Partly  Quite a bit  Completely | CPES-IC |
| Did you receive enough information about what to do if you were worried about your condition or treatment after you left the hospital? | Not at all  Partly  Quite a bit  Completely | CPES-IC |
| Before you left the hospital. You met with a member of your care team to review your discharge instructions and were given a sheet called PODS with instructions on it. Do you remember this? | Yes  No | Project team |
| Did having a PODS add value to the discharge experience? Why or why not? | Yes  No  Open comment field | Project team |

**Provider Survey Information**

| **Question** | **Response Options** | **Source** |
| --- | --- | --- |
| How often do you provide discharge teaching or the discharge summary/PODS form while the family caregiver or trusted other is present? | Scale from 0=never to 10=always  Open comment field | Project team |
| How often do you use teach-back or a similar patient engagement strategy when providing discharge teaching? | Scale from 0=never to 10=always  Open comment field | Project team |
| Did providing a PODS add to your workload? | Yes  No  Open comment field | Project team |
| Did providing a PODS add value to the discharge experience? | Yes  No  Open comment field | Project team |
